# SAAV2152 is a single-stranded DNA binding protein: the third SSB in *Staphylococcus aureus*

**DOI:** 10.18632/oncotarget.24427

**Published:** 2018-02-05

**Authors:** Yen-Hua Huang, Cheng-Yang Huang

**Affiliations:** ^1^ School of Biomedical Sciences, Chung Shan Medical University, Taichung City, Taiwan; ^2^ Department of Medical Research, Chung Shan Medical University Hospital, Taichung City, Taiwan

**Keywords:** SsbA, ssDNA, PriA, NSC5426, novel SSB, Immunology

## Abstract

Single-stranded DNA-binding proteins (SSBs) play crucial roles in DNA replication, repair, and recombination. Unlike *E. coli*, which contains only one type of SSB (EcSSB), some bacteria have two paralogous SSBs, namely, SsbA and SsbB. In this study, we found the third SSB-like protein in *Staphylococcus aureus*, SAAV2152, which was designated as SaSsbC. SaSsbC is a protein of 131 amino acids and shares 38%, 36%, and 33% sequence identity to SaSsbB, SaSsbA, and EcSSB, respectively. Gene map analysis showed that unlike the *E. coli ssb* gene, which is adjacent to *uvrA* gene, the *S. aureus ssb* gene *SAAV2152* is flanked by the putative *SceD*, the putative *YwpF*, and *fabZ* genes. A homology model showed that SaSsbC consists of the classic oligonucleotide/oligosaccharide-binding fold at the N-terminus. At the C-terminus, SaSsbC did not exhibit sequence similarity to that of EcSSB. Electrophoretic mobility shift analysis showed that SaSsbC formed a single complex with ssDNA of different lengths. Mutational analysis revealed that Tyr36, Tyr47, Phe53, and Tyr81 in SaSsbC are at positions that structurally correspond to the important residues of EcSSB for binding to ssDNA and are also critical for SaSsbC to bind ssDNA. Unlike EcSSB, which can stimulate EcPriA, SaSsbC did not affect the activity of SaPriA. In addition, SaSsbA inhibitor 9-methyl-2,3,7-trihydroxy-6-fluorone (NSC5426) could inhibit the ssDNA-binding activity of SaSsbC with IC_50_ of 78 μM. In conclusion, this study has identified and characterized SAAV2152 as a kind of SSB, and further research can directly focus on determining its actual physiological role in *S. aureus*.

## INTRODUCTION

Single-stranded DNA-binding proteins (SSBs) play crucial roles in DNA metabolic processes, such as DNA replication, repair, and recombination in both prokaryotes and eukaryotes [1, 2]. During these reactions, SSB is required to maintain the transient unwinding of duplex DNA in the single-stranded state. SSB binds and protects susceptible ssDNA from nucleases and chemical attacks [3]. SSB binds to ssDNA with high affinity regardless of sequence. Four distinct DNA-binding domains in SSBs have been identified, namely, the oligonucleotide/oligosaccharide-binding fold (OB fold), the K homology domain, the RNA recognition motif, and the whirly domain [4]. Most but not all bacterial SSBs are active as homotetramers, in which four OB folds form a DNA-binding domain [5, 6]. SSB from the bacterial phylum *Deinococcus*-*Thermus* functions as a homodimer, in which each monomer contains two OB folds linked by a conserved spacer sequence [7, 8]. In addition to DNA binding, SSB also binds to many DNA-binding proteins that constitute the SSB interactome [2, 9, 10].

The functions of SSB have been studied extensively in *Escherichia coli*. *E. coli* SSB (EcSSB) has two major ssDNA binding modes [11]. The binding mode is dependent on the concentrations of protein and salt in the solution. EcSSB consists of an N-terminal ssDNA-binding/oligomerization domain and a flexible C-terminal protein–protein interaction domain (SSBc) [2, 4]. SSBc can be further subdivided into two sub-domains, as follows: a long proline- or glycine-rich hinge, also known as the intrinsically disordered linker; and the highly conserved acidic tail of the last six C-terminal amino acid residues of SSB (DDDIPF) [2, 12]. This C-terminal acidic tail of SSB is essential for binding to more than a dozen different proteins [2] and can stimulate the activities of some of these proteins [13, 14]. SSBc, not only the C-terminal acidic tail, can also interact with the OB fold and regulate the ssDNA-binding activity of SSB itself [15, 16].

Unlike *E. coli*, which contains only one type of SSB, several bacteria have two paralogous SSBs, namely, SsbA and SsbB [17–19]. SsbA shares strong sequence similarity with the N-terminal DNA-binding domain and the C-terminal acidic tail of SSB and is thus referred to as a counterpart of EcSSB. *Bacillus subtilis* SsbA (BsSsbA) is a protein of 172 aa, essential for genome maintenance [20]. In contrast to BsSsbA, the 113-aa-protein BsSsbB is specialized for transformational recombination. Although crystal structures suggest that SsbA binds ssDNA in a manner similar to SsbB [21, 22], their DNA-binding properties are different. BsSsbB binds to ssDNA with lesser affinity than BsSsbA, whereas *Streptomyces coelicolor* SsbB (ScSsbB) exerts greater DNA-binding affinity than ScSsbA [21–23]. In addition, BsSsbB and ScSsbB but not *Streptococcus pneumonia* SsbB (SpSsbB), lack the C-terminal acidic tail of SSB for protein–protein interactions. Thus, SsbBs from different organisms may exhibit different protein–protein interaction specificities.

PriA helicase is utilized during replication restart to reload the replicative DnaB helicase back onto the chromosome [24–26]. Fuelled by the binding and hydrolysis of ATP, PriA acts with other primosomal proteins and separates double-stranded DNA (dsDNA) into their complementary single strands [27–29]. On its own, PriA is a poor helicase and might need other accessory proteins, such as PriB and SSB, to stimulate helicase activity [13, 14]. However, the main SSB of *Staphylococcus aureus* (SaSsbA) does not stimulate SaPriA [30]. Only SaDnaD enhances the ATPase activity of SaPriA [31]. Thus, the manner by which SaSsbA and SaSsbB participate in the SaPriA-directed primosome assembly and in the DNA replication restart process is still unclear.

In this study, we found and identified the third SSB-like protein in S. aureus. This novel SSB, designated as SaSsbC, has been cloned, overexpressed, and biochemically characterized. Results from the sequence alignment, structural modeling, and mutational analyses indicated a similar ssDNA-binding mode between SaSsbC and EcSSB. Although the C-terminal domain of SaSsbC exhibits no sequence similarity with that of EcSSB, SaSsbC is a typical SSB protein in many aspects.

## RESULTS

### The third *ssb* gene

**Figure 1:**
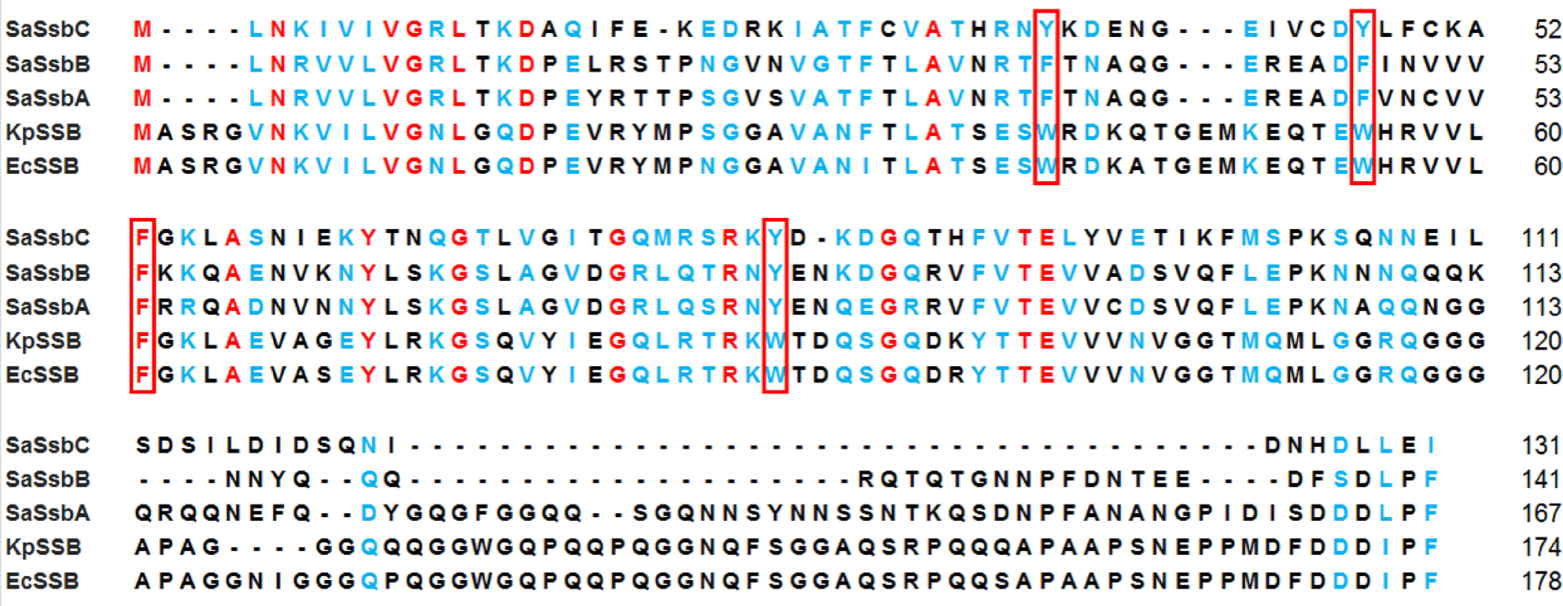
Multiple amino acid sequence alignment of SSB proteins Sequence alignment of SaSsbC, SaSsbB, SaSsbA, KpSSB, and EcSSB was generated by CLUSTALW2. Identical amino acid residues are colored in red. Amino acid residues with similar properties judged by CLUSTALW2 are colored in light blue. The essential aromatic residues involved in ssDNA binding are boxed. The C-terminal domains of these SSBs are not conserved.

Some bacteria have two paralogous SSBs (SsbA and SsbB) [19]. They have similar nucleotide sequence with *E. coli* SSB. Three *S. aureus* ssb genes (*SAAV0334*, *SAAV0835*, and *SAAV2152*) were found after searching through the National Center for Biotechnology Information (NCBI). These three SSBs share an overall 36% sequence identity and are mostly conserved in the first 110 aa (i.e. the N-terminal domain). Based on similarity with BsSsbA and BsSsbB, *SAAV0334* encodes SaSsbA of 167 aa, whereas *SAAV0835* encodes SaSsbB of 141 aa. *SAAV2152* encodes a protein of 131 aa. This SSB was designated as SaSsbC in this study (Figure 1).

### Sequence analysis of SaSsbC

**Figure 2:**
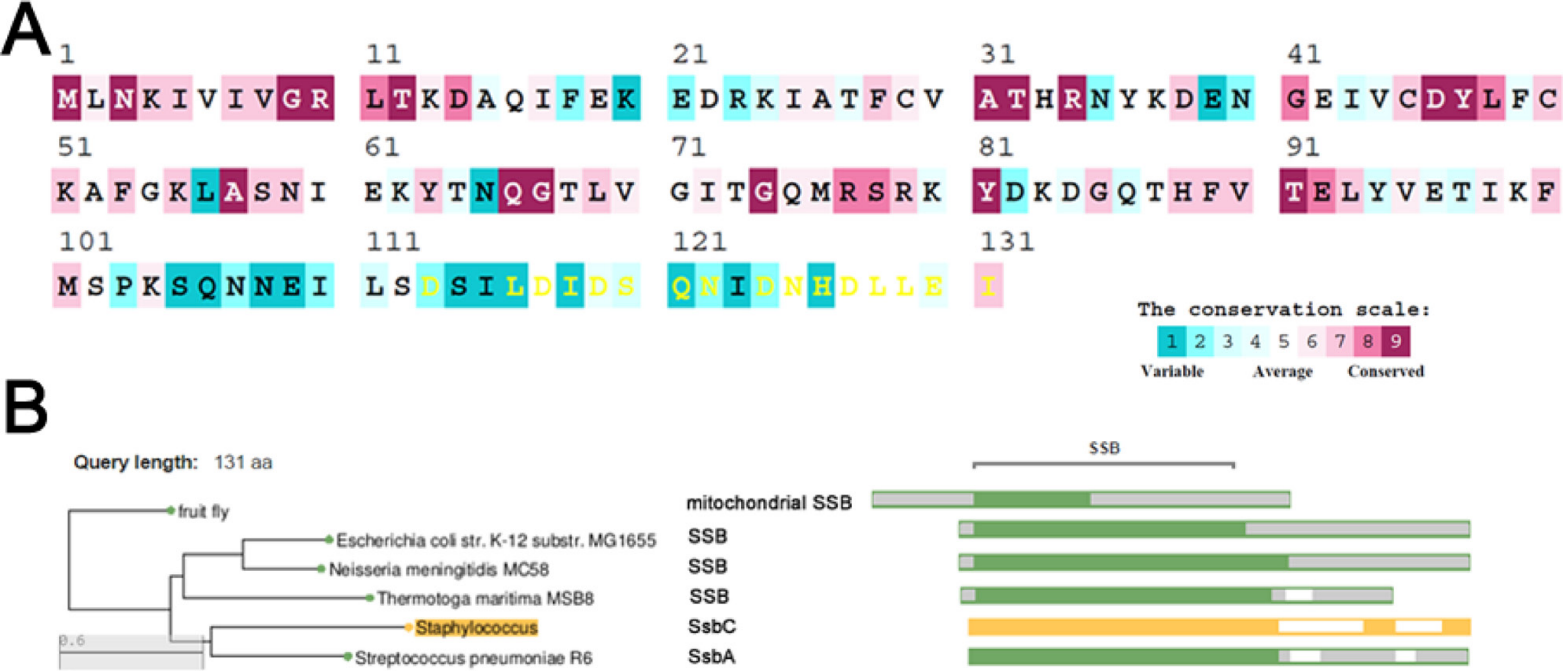
Sequence analysis of SaSsbC (**A**) An alignment consensus of 698 sequenced SSB homologs by ConSurf reveals the degree of variability at each position along the primary sequence. Highly variable amino acid residues are colored teal, whereas highly conserved amino acid residues are burgundy. A consensus sequence was established by determining the most commonly found amino acid residue at each position relative to the primary sequence of SaSsbC. (**B**) The evolutionary tree of SSBs with SaSsbC was generated by SmartBlast. They could be classified into at least 6 groups of SSB family.

The amino acid sequence of SaSsbC shares 38%, 36%, 33%, and 32% identity to that of SaSsbB, SaSsbA, EcSSB, and KpSSB, respectively (Figure 1), with SaSsbC being the shortest one. Analysis of SaSsbC by RPS-BLAST showed the presence of a putative OB-fold domain that is common to all known SSBs. Figure 2A shows the alignment consensus of 698 sequenced SSB homologs by ConSurf [32], revealing the degree of variability at each position along the primary sequence. The amino acid residues in the C-terminal region of SaSsbC are variable. In the EcSSB–ssDNA complex [5], four essential aromatic residues, Trp40, Trp54, Phe60, and Trp88, conserved in most SSB families as Phe/Tyr/Trp, participate in ssDNA binding via stacking interactions. The corresponding residues in SaSsbC are Tyr36, Tyr47, Phe53, and Tyr81. No Trp residue was observed in SaSsbC (Figure 1). The protein sequence analogous to the C-terminal tail DDDIPF in EcSSB involved in protein–protein interaction is HDLLEI in SaSsbC. Thus, similar to ScSsbB [21], SaSsbC lacks the acidic tail that is conserved in all main SSB sequences. The evolutionary tree for SSBs with SaSsbC showed that they could be classified into at least 6 groups (Figure 2B). Although SaSsbC and SpSsbA share 34% identity, they were classified into different phylogenetic groups by SmartBlast.

### Analysis of the *ssb* gene *SAAV2152*

**Figure 3:**
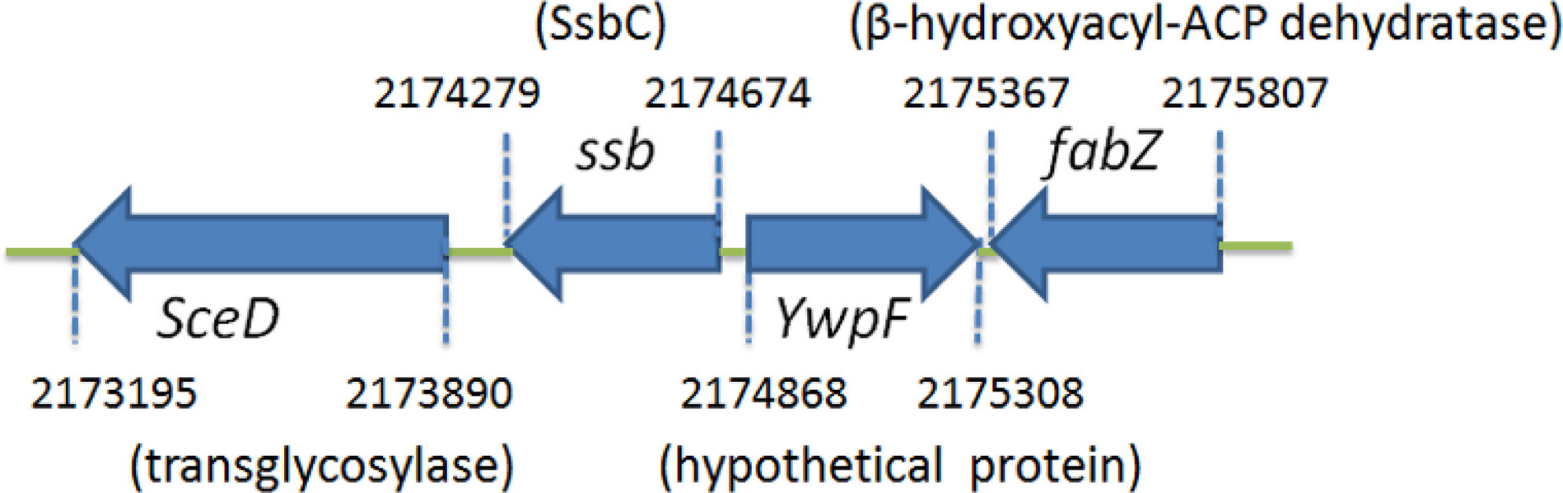
Gene map of *S. aureus* chromosomal region with the *ssb* gene *SAAV2152* The gene *SAAV2152* coding for SaSsbC maps from the 2174279 to 2174674 nt of the *S. aureus* genome. *This ssb* gene is flanked by the putative *SceD*, the putative *YwpF*, and *fabZ* genes, coding for a transglycosylase (231 aa), a hypothetical protein (167 aa), and a β-hydroxyacyl-ACP dehydratase (146 aa), respectively.

Figure 3 shows the gene map of *S. aureus* chromosomal region with the third *ssb* gene, which is flanked by the putative *SceD,* the putative *YwpF*, and *fabZ* genes, which code for a transglycosylase (231 aa), a hypothetical protein (167 aa), and a β-hydroxyacyl-ACP dehydratase (146 aa), respectively. *S. aureus* and *B. subtilis* main *ssb* genes are flanked by *rpsF* and *rpsR* and are controlled by the SOS response [19], whereas the gene regulation of the *ssb* gene *SAAV2152* coding for SaSsbC is almost unknown.

### Purification of SaSsbC

*SAAV2152* coding for the putative SSB-like protein *designated as SaSsbC in this study* was PCR-amplified using the genomic DNA of *S. aureus* subsp. *aureus* ED98 as template. This amplified gene was then ligated into the pET21b vector for protein expression. SaSsbC with a His tag was heterologously overexpressed in *E. coli* and then purified from the soluble supernatant by Ni^2+^-affinity chromatography. Pure protein was obtained in this single chromatographic step with an elution of Buffer A and dialyzed against a dialysis buffer (Buffer B). Approximately >15 mg of purified protein was obtained from 1 L of *E. coli* cell culture.

### SaSsbC bound to ssDNA

**Figure 4:**
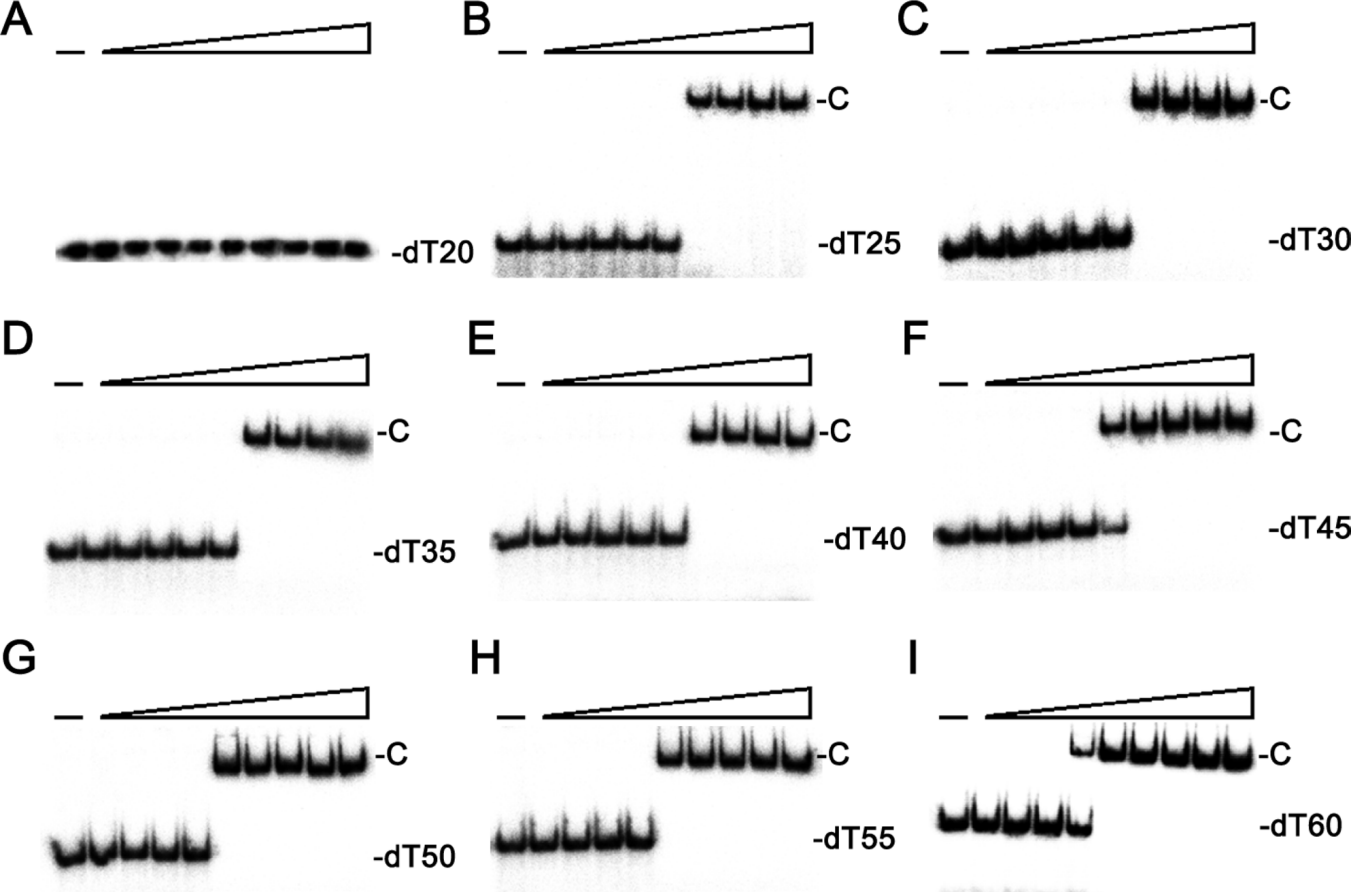
EMSA of SaSsbC Protein (0, 0.01, 0.02, 0.039, 0.078, 0.1563, 0.3125, 0.625, 1.25, and 2.5 μM; tetramer) was incubated with 1.7 nM of (**A**) dT20, (**B**) dT25, (**C**) dT30, (**D**) dT35, (**E**) dT40, (**F**) dT45, (**G**) dT50, (**H**) dT55, or (**I**) dT60, and then analyzed by EMSA.

**Figure 5:**
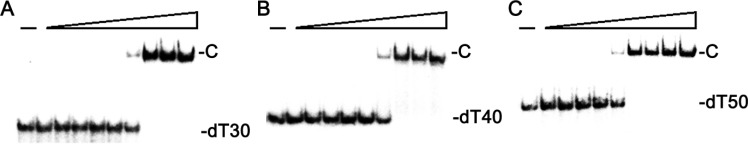
EMSA of SaSsbC in the presence of 0.4 M NaCl Protein (0, 0.01, 0.02, 0.039, 0.078, 0.1563, 0.3125, 0.625, 1.25, and 2.5 μM; tetramer) was incubated with 1.7 nM of (**A**) dT30, (**B**) dT40, or (**C**) dT50 in 20 mM Tris—HCl (pH 8.0) and 400 mM NaCl. The resultant solution was then analyzed by EMSA.

Given that the sequence analysis may indicate SaSsbC as a kind of SSB, we attempted to test whether SaSsbC has ssDNA-binding activity. We studied the binding of SaSsbC to ssDNA of different lengths with different protein concentrations using EMSA (Figure 4). EMSA is a well-established approach in studies of molecular biology, allowing the detection of the distinct protein–DNA complex(es) [33]. When we incubated SaSsbC with dT20, no significant band shift was observed (Figure 4A), indicating that SaSsbC could not form a stable complex with dT20. We further tested dT25–60 to bind to SaSsbC. In contrast with dT20, longer dT homopolymers, such as dT25–60, produced a very significant band shift (Figure 4; C, complex). These findings confirm the ssDNA-binding activity of SaSsbC, which was strong enough to form a stable protein–DNA complex in solution. The binding ability of SaSsbC to dT20, dT30, and dT40 in the presence of 0.4 M NaCl was also analyzed (Figure 5). To compare the binding abilities of SaSsbC with ssDNA of different lengths, as well as the salt effect on the ssDNA-binding abilities of SaSsbC, we calculated the midpoint values for input ssDNA binding from the titration curves of EMSA, and the [Protein]_50_ values were quantified using linear interpolation from the protein concentration (Table 1). The ssDNA-binding ability of SaSsbC correlated with the length of ssDNA, that is, with longer ssDNA corresponded to higher binding affinity. In addition, salt suppressed the binding of SaSsbC to ssDNA. For example, The [SaSsbC]_50_ for dT40 binding was 225 ± 15 nM, which was about twofold lower than in the presence of 0.4 M NaCl (418 ± 26 nM).

**Table 1:** The [Protein]_50_ values of SaSsbC as analyzed by EMSA

DNA	[Protein]_50_ (nM)
dT20	ND
dT25	234 ± 18
dT30	230 ± 8
dT30 (with 0.4 M NaCl)	420 ± 26
dT35	220 ± 16
dT40	225 ± 15
dT40 (with 0.4 M NaCl)	418 ± 26
dT45	125 ± 9
dT50	117 ± 10
dT50 (with 0.4 M NaCl)	208 ± 18
dT55	115 ± 7
dT60	102 ± 6
PS4/PS3	ND
PS4/PS3–dT5	ND
PS4/PS3–dT10	ND
PS4/PS3–dT15	ND
PS4/PS3–dT20	ND
PS4/PS3–dT25	232 ± 18
M1/M2-M3	190 ± 18
S1/M2-M3	ND

[Protein]_50_ was calculated from the titration curves of EMSA by determining the concentration of the protein (tetramers) needed to achieve the midpoint value for input DNA binding. Errors are standard deviations determined by three independent titration experiments.

### Oligomeric state of SaSsbC in solution

**Figure 6:**
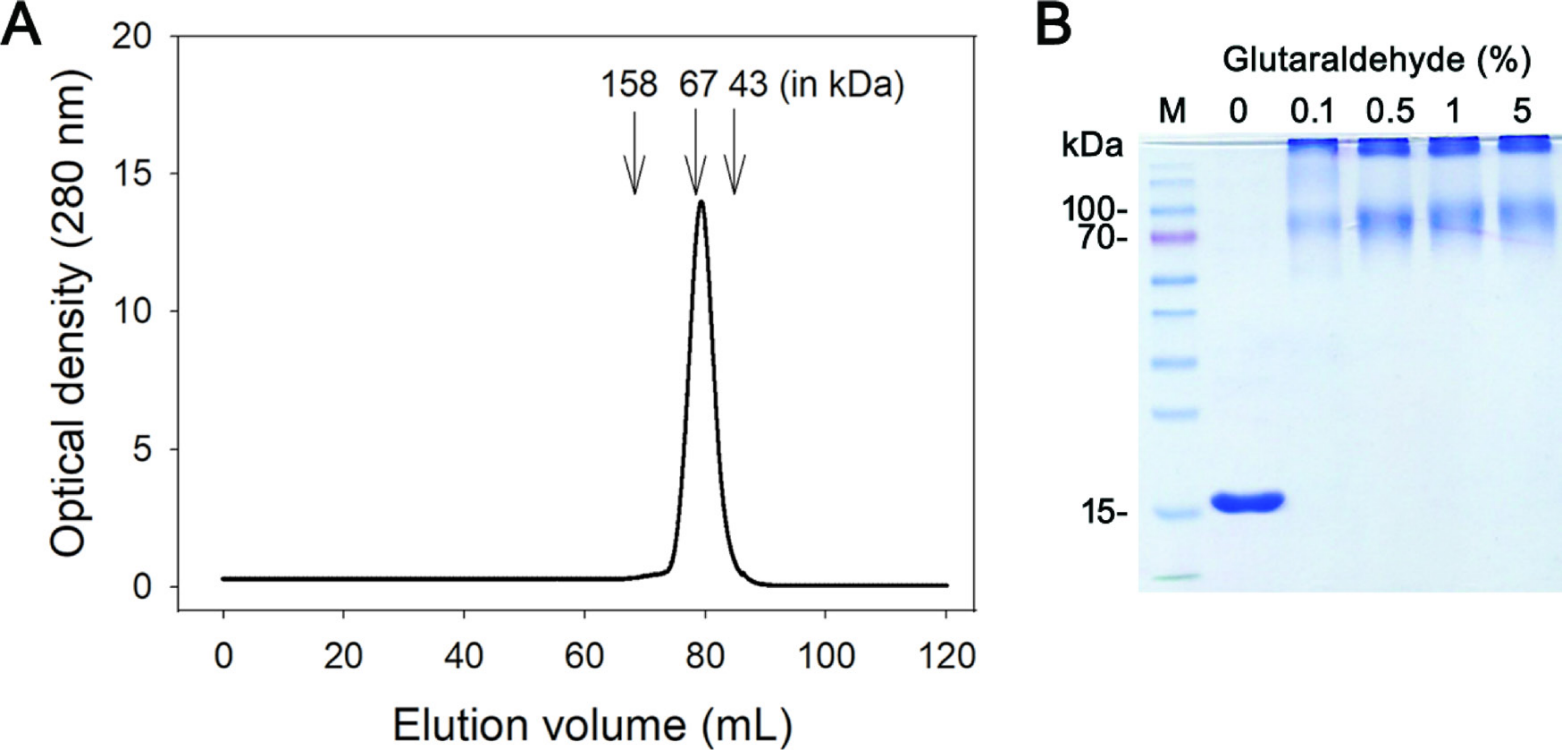
Oligomeric state of purified SaSsbC in solution (**A**) Gel filtration chromatographic analysis (Superdex 200 prep grade column) of purified SaSsbC. The column was calibrated with proteins of known molecular masses: thyroglobulin (670 kDa), γ-globulin (158 kDa), albumin (67 kDa), ovalbumin (43 kDa), chymotrypsinogen A (25 kDa) and ribonuclease A (13.7 kDa). The corresponding peak shows the eluted SaSsbC. (**B**) Glutaraldehyde cross linking of SaSsbC. SaSsbC (2.5 μM) was incubated with increasing concentrations of glutaraldehyde (0.1% to 5%) at 4° C for 30 min. Coomassie Blue-stained SDS-PAGE of the resulting samples and molecular mass standards are shown.

The oligomeric state of purified SaSsbC was analyzed by gel filtration chromatography (Figure 6A). The native molecular mass of SaSsbC was estimated to be 64 kDa, which is approximately four times the molecular mass of a SaSsbC monomer (15 kDa). Thus, SaSsbC in solution is a stable tetramer like SaSsbA [30], EcSSB [5], PaSSB [34], StSSB [35], and KpSSB [36].

To further substantiate the observation made from gel filtration chromatography, chemical cross-linking of SaSsbC using glutaraldehyde was performed (Figure 6B). 2.5 μM SaSsbC was incubated with increasing concentrations of glutaraldehyde (0.1%–5%) at 4° C for 30 min. At these concentrations, the tetrameric form of SaSsbC was observed. The glutaraldehyde cross-linking result showed that SaSsbC occurred as a tetramer in solution, consistent with that analyzed using gel filtration chromatography.

### SaSsbC cannot bind dsDNA

**Figure 7:**
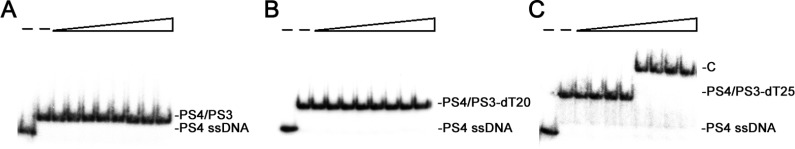
Binding analysis of SaSsbC to dsDNA Protein (0, 0, 0.02, 0.039, 0.078, 0.1563, 0.3125, 0.625, 1.25, and 2.5 μM; tetramer) was incubated with 1.7 nM of (**A**) PS4/PS3, (**B**) PS4/PS3–dT20, or (**C**) PS4/PS3–dT25.

Sequence similarity (Figure 1) and ssDNA-binding analysis (Figure 4) indicated that SaSsbC is a type of SSB, similar to SaSsbA and EcSSB. To investigate whether SaSsbC binds dsDNA, the 25-bp dsDNA substrate for EMSA was prepared by annealing oligonucleotides PS4 and PS3, of which one DNA strand was radiolabeled. No band shift was observed when SaSsbC was incubated with PS4/PS3. The absence of band shift indicated that SaSsbC could not form a stable complex with this DNA substrate during electrophoresis (Figure 7A). We further tested whether SaSsbC binds to dsDNA with ssDNA overhang of 5–25 mer dT. SaSsbC could not form a stable complex with the following DNA substrates: PS4/PS3–dT5, PS4/PS3–dT10, PS4/PS3–dT15, and PS4/PS3–dT20 (Figure 7B). However, SaSsbC could form a stable complex with PS4/PS3–dT25 (Figure 7C). Given that SaSsbC can bind to ssDNA dT25 (Figure 4B) but not to dT20 (Figure 4A), we hypothesized that SaSsbC can likely bind to PS4/PS3–dT25 because of the dT25 tail in PS4/PS3–dT25. In addition, the [SaSsbC]_50_ for PS4/PS3–dT25 binding was 232 ± 18 nM, a value similar to that for dT25 binding (234 ± 18 nM). Thus, SaSsbC cannot bind to dsDNA on the basis of the EMSA results.

### SaSsbC can bind ssDNA-containing forked DNA

**Figure 8:**
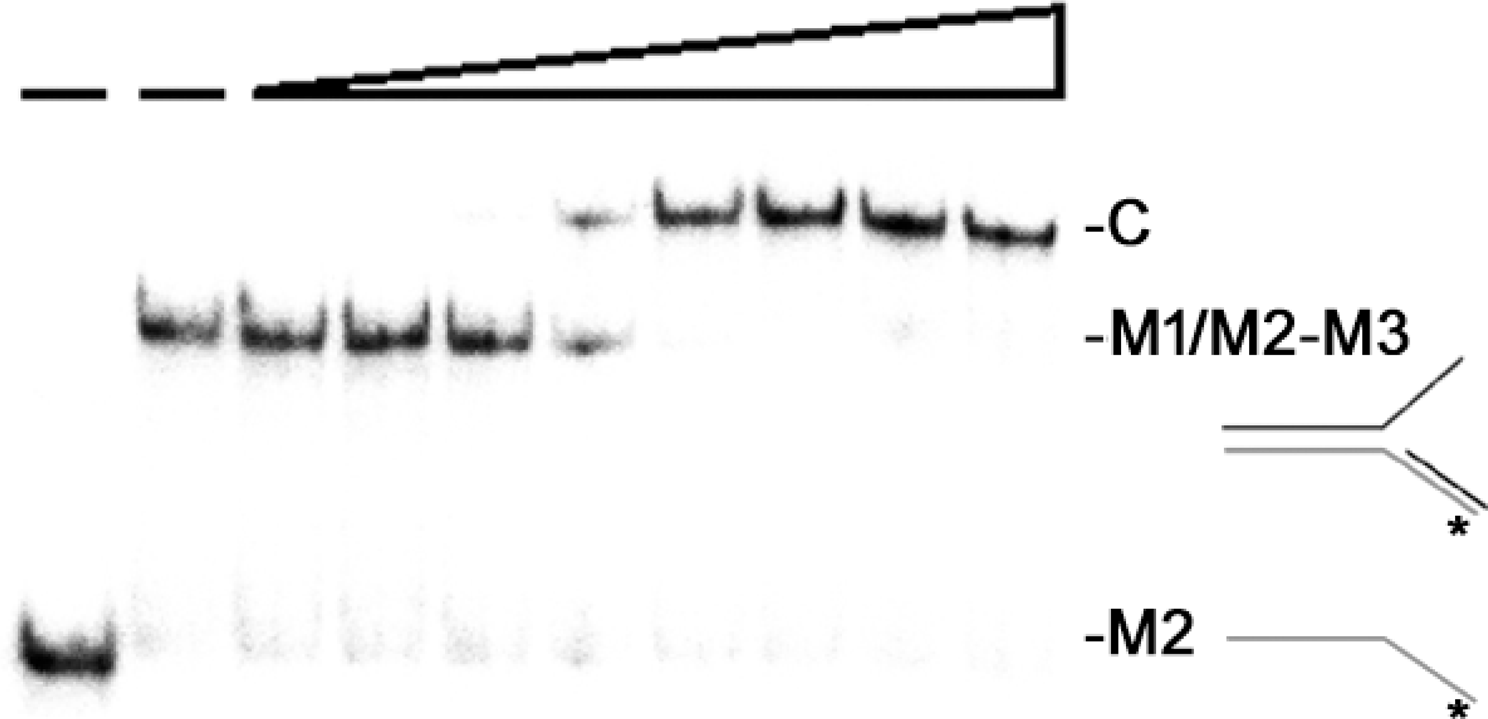
Binding analysis of SaSsbC to forked DNA Protein (0, 0, 0.02, 0.039, 0.078, 0.1563, 0.3125, 0.625, 1.25, and 2.5 μM; tetramer) was incubated with 1.7 nM M1/M2-M3 forked DNA substrate.

To investigate whether SaSsbC binds to forked DNA, we prepared M1/M2-M3 forked DNA substrate for EMSA by annealing oligonucleotides M1 (90 bp), M2 (78 bp), and M3 (28 bp), of which M2 was radiolabeled. This DNA substrate will produce a 40-bp ssDNA region when completely annealed. SaSsbC could form a stable complex with M1/M2-M3 forked DNA with [SaSsbC]_50_ of 190 ± 18 nM (Figure 8). This binding constant was slightly higher than that for dT40 homopolymer binding (225 ± 15 nM). We also tested whether SaSsbC binds to forked DNA with shorter ssDNA (S1/M2-M3; S1, 70 bp). This DNA substrate will produce a 20-bp ssDNA region when completely annealed. No band shift was observed when SaSsbC was incubated with S1/M2-M3 forked DNA (data not shown). Thus, we hypothesized that SaSsbC can likely bind to M1/M2-M3 forked DNA because of the 40-bp tail in this DNA.

### SaSsbC cannot stimulate the ATPase activity of SaPriA

**Figure 9:**
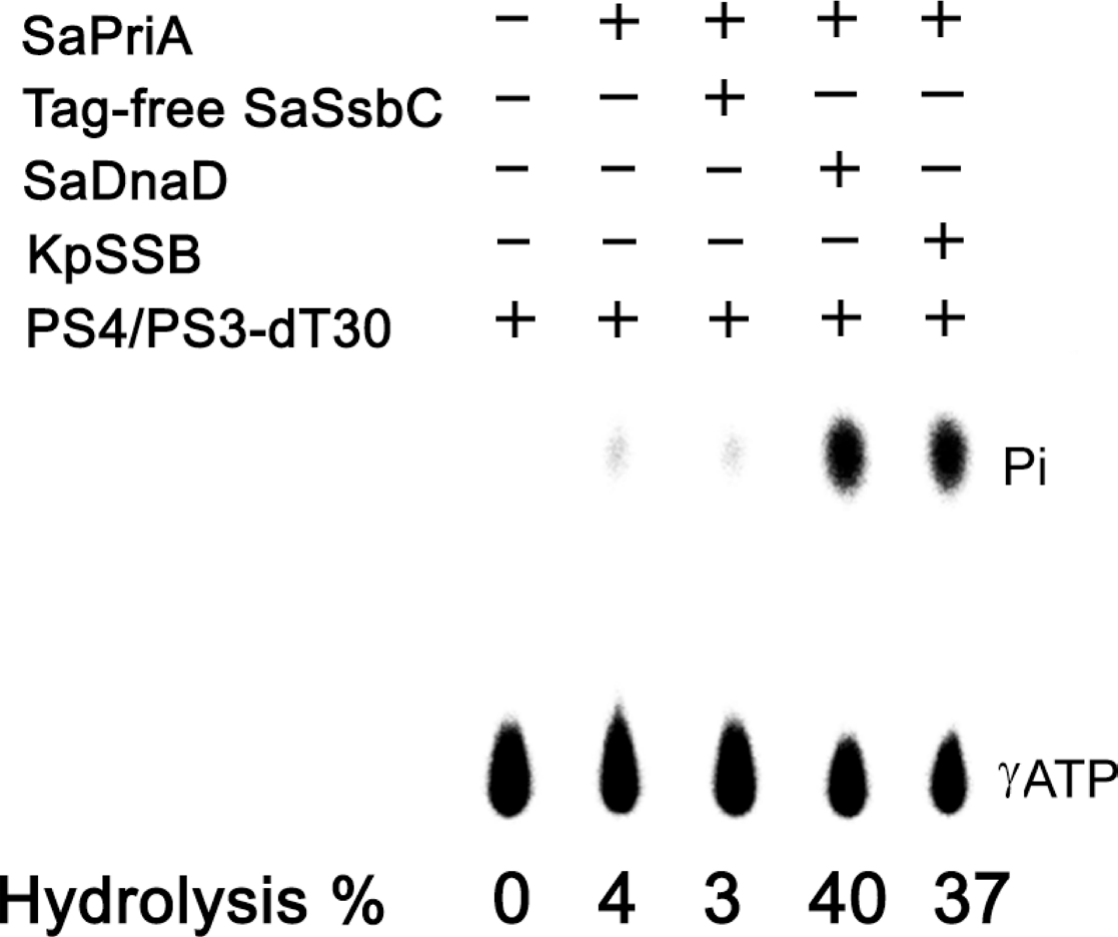
The ATPase activity of SaPriA did not change when acting with SaSsbC SaPriA ATPase assay was performed with 0.4 mM [γ-32P] ATP, 0.12 μM of SaPriA, and 0.1 μM PS4/PS3-dT30 DNA substrate for 1 h. To study the effect, tag-free SaSsbC (10 μM), KpSSB (10 μM), or SaDnaD (10 μM) was added into the assay solution. Aliquots (5 μL) were taken and spotted onto a polyethyleneimine cellulose thin-layer chromatography plate, which was subsequently developed in 0.5 M formic acid and 0.25 M LiCl for 30 m. Reaction products were visualized by autoradiography and quantified with a Phosphorimager.

On its own, PriA is a poor helicase *in vitro* [37]. Gram-negative EcPriA activity can be significantly stimulated by EcPriB and EcSSB [13, 14]. SaPriA activity can be stimulated by SaDnaD [31]; however, unlike EcSSB, SaSsbA cannot stimulate SaPriA [30]. Whether or not SaSsbC can enhance SaPriA activity is still unknown. To investigate the possible effect of SaSsbC, we performed ATPase assay for SaPriA. SaDnaD [31] and KpSSB [30], which are known to stimulate SaPriA activity, were used as positive controls. As shown in Figure 9, SaPriA could hydrolyze ATP on its own; however, no effect was found on SaPriA activity when acting with SaSsbC. To exclude the possible effect of a His tag, we used a tag-free SaSsbC for this assay (Figure 9). Thus, similar to SaSsbA, SaSsbC does not affect the activity of SaPriA.

### Structural modeling of SaSsbC

**Figure 10:**
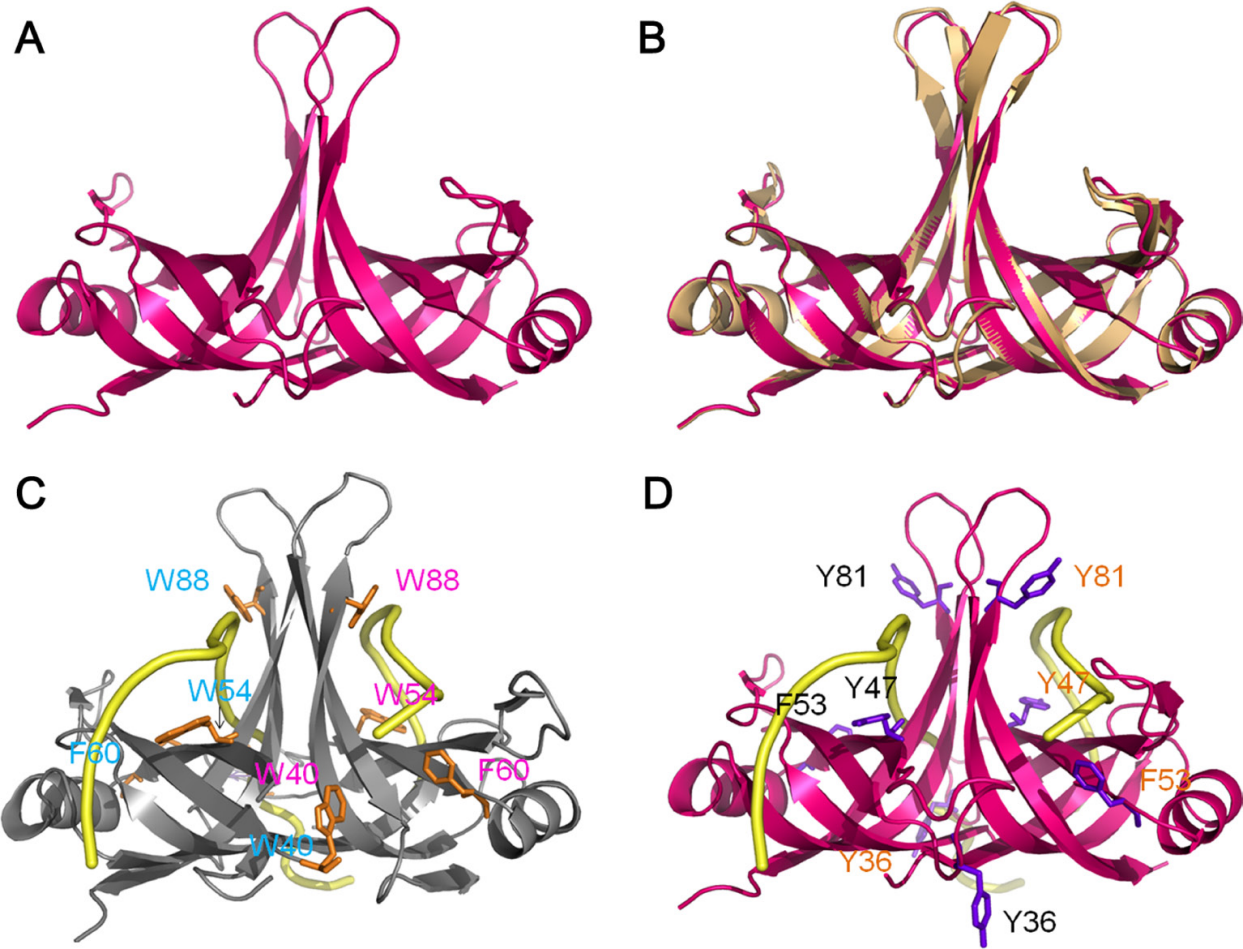
Structure modeling (**A**) Structure modeling of the N-terminal domain (aa 1-103) of SaSsbC by SWISS-MODEL using SaSsbA as a template. For clarity, only a dimer of SaSsbC is shown. (**B**) Superposition of SaSsbC and SaSsbA. The N-terminal domains of SaSsbC and SaSsbA (PDB entry 5GXT; wheat) are similar. The structural model of SaSsbC (aa 1–103) reveals an OB-fold domain similar to SaSsbA. (**C**) Complexed crystal structure of EcSSB. Four essential aromatic residues, Trp40, Trp54, Phe60, and Trp88, participate in ssDNA binding (PDB entry 1EYG; gray). (**D**) ssDNA-binding mode of SaSsbC. Based on the structural similarity between SaSsbC and EcSSB, the corresponding residues in SaSsbC are Tyr36, Tyr47, Phe53, and Tyr81, which may play a similar role in ssDNA binding as those in EcSSB.

The crystal structure of SaSsbC is yet to be determined. We modeled SaSsbC by homology modeling using SWISS-MODEL (http://swissmodel.expasy.org/) [38]. The N-terminal domain of SaSsbA (PDB entry 5GXT) [30] was the first hit suggested as a template by the program. The structural model of SaSsbC (aa 1–103) revealed an OB-fold domain (Figure 10A) similar to that of SaSsbA (Figure 10B) and EcSSB (Figure 10C), with the core of the OB-fold possessing a β-barrel capped by an α-helix. In the EcSSB–ssDNA complex (Figure 10C), four essential aromatic residues, Trp40, Trp54, Phe60, and Trp88, participate in ssDNA binding via stacking interactions [5]. The corresponding residues in SaSsbC are Tyr36, Tyr47, Phe53, and Tyr81, which may play a similar role in ssDNA binding as those in EcSSB (Figure 10D).

### Mutational analysis

**Figure 11:**
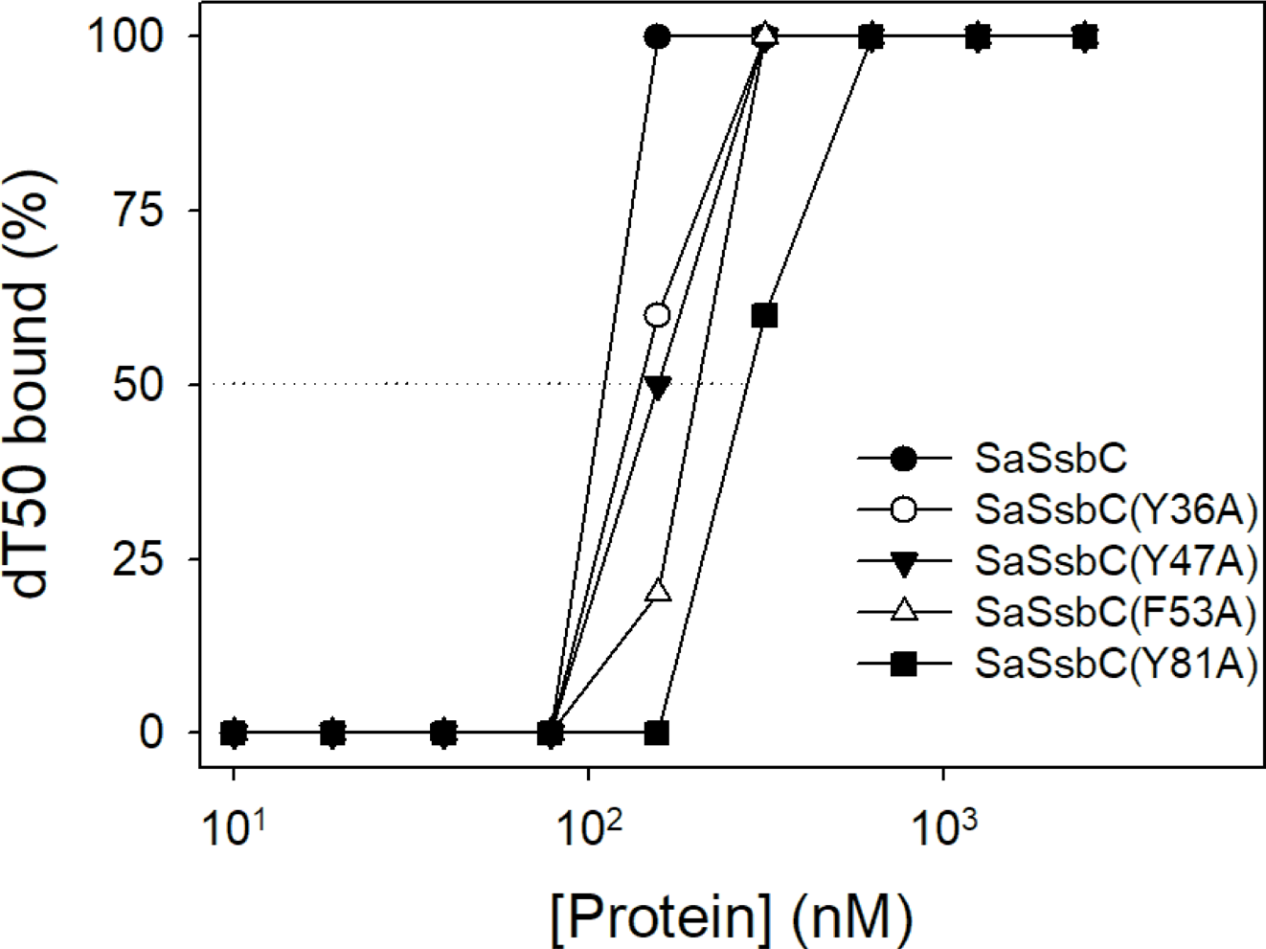
Mutational analysis of SaSsbC for ssDNA binding Binding of SaSsbC mutant protein (Y36A, Y47A, F53A, and Y81A) to dT50. The mutant protein (0, 0.01, 0.02, 0.039, 0.078, 0.1563, 0.3125, 0.625, 1.25, and 2.5 μM; tetramer) was incubated with 1.7 nM dT50.

On the basis of the proposed structural model of SaSsbC (Figure 10) and in comparison with the EcSSB-ssDNA complex structure, the four aromatic residues (i.e. Tyr36, Tyr47, Phe53, and Tyr81) located on the protein surface may be involved in ssDNA binding via stacking interactions. These residues in SaSsbC allow nucleic acids to wrap around the whole SaSsbC, similar to that in EcSSB-ssDNA complex [5]. According to the EcSSB-ssDNA complex structure, we manually superimposed the location of ssDNA with the structure model of SaSsbC (Figure 10D). To test whether the proposed model can possibly be used to form the SaSsbC–ssDNA complex, alanine substitution mutants (i.e., Y36A, Y47A, F53A, and Y81A) were conducted and analyzed by EMSA (Figure 11). The [Protein]_50_ values for the binding of these SaSsbC variants to dT50 are summarized in Table 2. These SaSsbC mutants have [Protein]_50_ values that were higher than that of the wild-type SaSsbC. The mutational effect on the ssDNA binding activity of SaSsbC followed the order Y81A > F53A > Y47A > Y36A; results of Y36A and Y47A were not very significant. Thus, SaSsbC may bind ssDNA in a manner similar to that in EcSSB.

**Table 2:** The [Protein]_50_ values of SaSsbC mutants as analyzed by EMSA

dT50	[Protein]_50_ (nM)
SaSsbC	117 ± 10
SaSsbC(Y36A)	139 ± 12
SaSsbC(Y47A)	157 ± 9
SaSsbC(F53A)	202 ± 26
SaSsbC(Y81A)	278 ± 18

[Protein]_50_ was calculated from the titration curves of EMSA by determining the concentration of the protein (tetramers) needed to achieve the midpoint value for input DNA binding. Errors are standard deviations determined by three independent titration experiments.

### Thermostability

**Figure 12:**
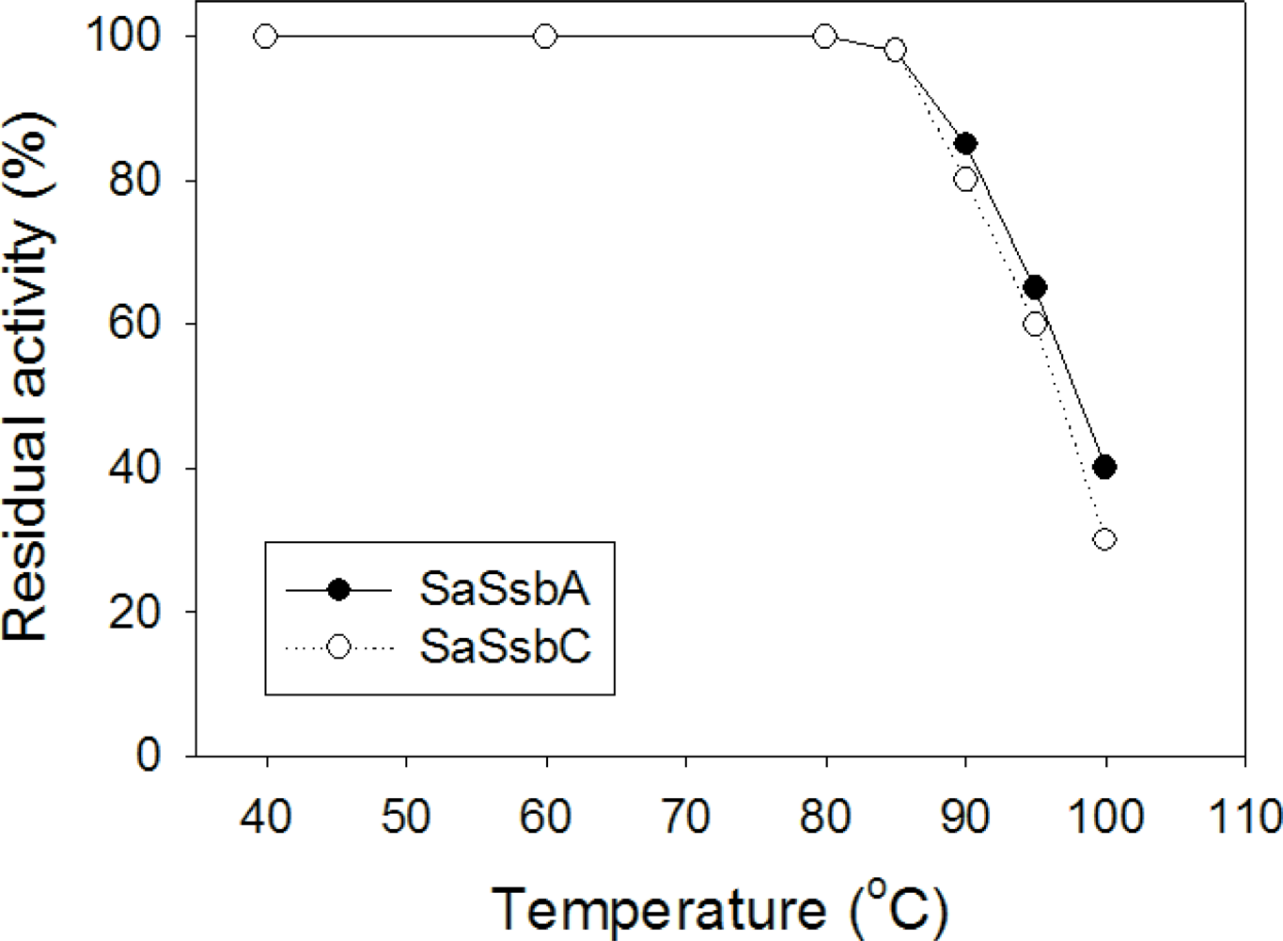
The thermostability of SaSsbC Protein (1 μM) was incubated at temperatures ranging from 40° C to 100° C for 30 min. The resultant protein solution was incubated at 25° C for 30 min with 1.7 nM dT30 in a total volume of 10 μL in 20 mM Tris–HCl (pH 8.0) and 100 mM NaCl. The phosphor storage plate was scanned, and the data for complex and free DNA bands were digitized for quantitative analysis.

SSB proteins have high thermostability even those coming from psychrophilic bacteria [39]. To investigate the stability of SaSsbC at elevated temperatures, we performed indirect thermostability experiments (Figure 12). The thermostability of SaSsbA was also analyzed. Incubation of SaSsbA and SaSsbC at 40, 60, and 80° C for 30 min showed no loss in binding activity to dT30. The activity of SaSsbA incubated for 30 min decreased by 60% at 100° C, 35% at 95° C, 15% at 90° C, and 2% at 85° C. The activity of SaSsbC incubated for 30 min decreased by 70% at 100° C, 40% at 95° C, 20% at 90° C, and 2% at 85° C. Given that the activity of EcSSB decreased by 50% after 30 min incubation at 95° C [39], we determined that the thermostability of these SSBs followed the order SaSsbA > SaSsbC > EcSSB. Thus, similar to EcSSB and other SSBs, SaSsbC exhibits high thermostability.

### SaSsbA inhibitor NSC5426 inhibits SaSsbC

**Figure 13:**
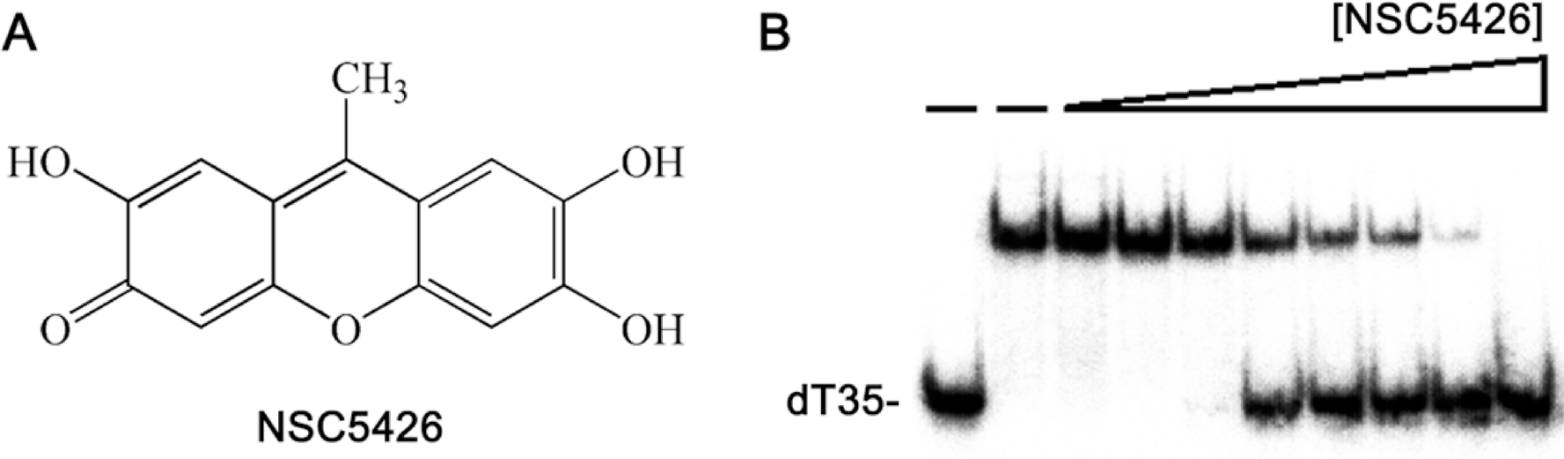
SaSsbA inhibitor NSC5426 inhibits SaSsbC (**A**) Molecular structure of NSC5426. (**B**) IC_50_ determination of NSC5426 for SaSsbC. Protein (0.3 μM; tetramer) was incubated with 1.7 nM dT35 and NSC5426 (0, 0, 9, 19, 39, 78, 156, 312, 625, and 1250 μM) at 25 °C for 30 min in a total volume of 10 μL in 20 mM Tris–HCl (pH 8.0) and 100 mM NaCl.

Some compounds are known to inhibit ssDNA-binding activity of SaSsbA [40]. For example, ssDNA-binding ability of SaSsbA can be completely suppressed by the presence of 100 μM 9-methyl-2,3,7-trihydroxy-6-fluorone (NSC5426) [40], a tricyclic planar compound (Figure 13A). The amino acid sequence of SaSsbC shares 36% identity with that of SaSsbA (Figure 1), particularly within the first 110 aa, which is the ssDNA-binding domain, and the model structure of SaSsbC resembles the crystal structure of SaSsbA possessing OB-folds (Figure 10). Thus, we tested whether SaSsbC could be inhibited by NSC5426, similar to SaSsbA. As shown in Figure 13B, NSC5426 can significantly inhibit SaSsbC binding to dT35 (Figure 13B). The IC_50_ value of SaSsbC for NSC5426, that is, the inhibitor concentration required to reduce the binding activity of the protein by 50%, was 78 ± 14 μM. Thus, SaSsbA inhibitor NSC5426 also inhibits SaSsbC.

We also tested whether or not the ssDNA-binding ability of SaSsbC can be inhibited by kaempferol, a flavonol with inhibitory activity on DnaB helicase binding to ssDNA [41]. Kaempferol also inhibits ssDNA-dependent ATPase activity of PriA helicase and DnaB helicase [41–43]. In the presence of 50 μM kaempferol, SaSsbC still bound to dT35 well (data not shown). Because of poor solubility of kaempferol, we could not conduct a test for possible inhibitory effect at higher concentrations. Thus, kaempferol did not reach the level for sufficient inhibition of SaSsbC.

## DISCUSSION

The discovery of small-molecule antibiotics for clinical use has been a seminal event in the field of infectious diseases [44]. *S. aureus*, a Gram-positive pathogen, exhibits a remarkable ability to develop antibiotic resistance, and few therapies are effective against methicillin-resistant *S. aureus* (MRSA) [45]. Considering that SSB is essential for all DNA-dependent cellular processes and that nucleic acid metabolism is one of the most basic biological functions, SSB should be a prime target in antibiotic development [46, 47]; therefore, SSBs become promising targets in antibiotic development [40]. Unlike *E. coli*, which produces only one type of SSB, *S. aureus* was indicated to express at least three SSBs in this study. SaSsbC, as well as SaSsbA and SaSsbB [40], may be a potential target against *S. aureus* growth and viability.

*In silico* analysis of the whole genome of S. aureus revealed the presence of another SSB-like protein in addition to SaSsbA and SaSsbB (Figure 3). These three SSBs in *S. aureus* share an overall 36% sequence identity (Figure 1), mostly being conserved in the first 110 aa, which is the N-terminal ssDNA-binding domain. In this study, we identified SaSsbC as a kind of SSB (Figure 4); however, its actual physiological role is yet to be understood. Gene map analysis showed that unlike *E. coli ssb* gene located adjacent to *uvrA* gene, the SaSsbC gene was shown to be flanked by the putative *SceD*, the putative *YwpF*, and *fabZ* genes, which code for a transglycosylase (231 aa), a hypothetical protein (167 aa), and a β-hydroxyacyl-ACP dehydratase (146 aa), respectively (Figure 3). If these genes belong to one operon or one regulatory control, SaSsbC may be regulated with these enzymes, which are involved in glycosidic linkages and fatty acid synthesis. However, the promoter for these genes is still unknown, and this hypothesized relationship must be further confirmed by a detailed transcription analysis.

SaSsbC exhibited a clear relationship with SSB at the amino acid sequence level, thereby suggesting that SaSsbC is a typical SSB protein in many respects. Results from the structural modeling (Figure 10) and mutational analyses (Figure 11) further indicated a similar ssDNA-binding mode between SaSsbC and EcSSB. Typical SSBs consist of an N-terminal OB-fold domain, a long proline/glycine-rich flexible region, and a C-terminal acidic tail. The three SSBs in *S. aureus* also comprised an N-terminal OB-fold domain and a long flexible region, but their flexible regions contained very few proline and glycine residues. In addition, SaSsbC and SaSsbB showed no C-terminal acidic peptide tail (Figure 1), which interacts with the partner protein. SSB binds many DNA-binding proteins via the PXXP motifs and the C-terminal acidic peptide tail such as RecG and PriA that constitute the SSB interactome [1, 2, 9, 10, 48, 49]. The lack of the acidic peptide tail and PXXP motifs in SaSsbB and SaSsbC may rule out the possibility of binding to SaPriA. Thus, no stimulation occurred (Figure 9). We speculate that SsbC may play a different physiological role from the main SSB.

Unlike OB-fold protein PriB [50, 51], which can bind dsDNA and ssDNA comparably [52], SaSsbC was demonstrated to bind dsDNA only when these DNA substrates contain ssDNA tail longer than 25 mers (Figure 7). Structurally, the N-terminal DNA binding domain of SaSsbC resembled PriB, in which the only significant difference was in the lengths of the β4 and β5 sheets. Whether the length of the β4 and β5 sheets in OB-fold proteins determines the ssDNA/dsDNA binding preference of SSB remains to be investigated. Many SSB proteins bind to ssDNA with some degree of positive cooperativity [11]. In this study, we found differing EMSA behaviors between SaSsbC and SSB proteins. SSB proteins form multiple distinct complexes with ssDNA of different lengths [12, 34–36, 53, 54], whereas SaSsbC binding to ssDNA dT25–dT60 formed only a single complex (Figure 4). Salt suppressed the binding of SaSsbC to ssDNA but did not change the complex number (Figure 5). The C-terminus in SSB can also interact with the OB fold and regulate the ssDNA-binding activity of SSB itself [15, 55]. Thus, further studies are needed to determine whether the C-terminal domain of SaSsbC, the most different between these SSB proteins, can change the EMSA patterns.

Flavonoids are the most common group of plant polyphenols with antioxidant, antiradical, anticancer, and antibacterial properties [56]. Some flavonoids are ATPase-inhibiting agents [42, 43]. Flavonoid derivatives have been developed as therapeutic agents for cancer [57]. In this study, we found that the flavonol kaempferol can inhibit the ssDNA-binding activity of DnaB helicase [41] but not that of SaSsbC. NSC5426, an inhibitor on ssDNA-binding ability of SaSsbA, can also inhibit the activity of SaSsbC with the IC_50_ value of 78 μM. Thus, NSC5426 may be a competent “dirty drug”, that is, a multi-target drug [58] against *S. aureus*. More studies are still needed for drug optimization and finding a new key target in *S. aureus* for antibiotic development.

## MATERIALS AND METHODS

### Construction of plasmids for SaSsbC, tag-free SaSsbC, KpSSB, SaSsbA, SaDnaD, and SaPriA expression

Construction of the SaSsbA [30], SaDnaD [31], SaPriA [42], and *Klebsiella pneumoniae* SSB (KpSSB) [36] expression plasmids has been reported. *SAAV2152*, the gene encoding a putative SSB-like protein (designated as SaSsbC), was amplified by PCR using the genomic DNA of *S. aureus* subsp. *aureus* ED98 as template. The forward and reverse primers were designed to introduce unique restriction sites, permitting the insertion of the amplified gene into the pET21b vector (Novagen Inc., Madison, WI, USA) for protein expression in *E. coli*. To obtain His tag-free SaSsbC, a fragment containing the coding sequence of SaSsbC and the stop codon was directly amplified and ligated into the pET21b vector. Primers used for construction of these plasmids are summarized in Table 3.

**Table 3:** Primers used for construction of plasmids

Oligonucleotide	Primer
SaSsbC-NdeI-N	GGGAACATATGCTAAATAAAATCGTAA
SaSsbC-XhoI-C	CCATTCTCGAGAATTTCTAATAAGTCA
Tag-free SaSsbC-NdeI-N	GGGAACATATGCTAAATAAAATCGTAA
Tag-free SaSsbC-XhoI-C	CCATTCTCGAGTTAAATTTCTAATAAGTCA
SaSsbC(Y36A)-N	TGTTGCAACGCACCGAAATGCTAAAGATGAAAATGGAGAA
SaSsbC(Y36A)-C	GATTTCTCCATTTTCATCTTTAGCATTTCGGTGCGTTGCA
SaSsbC(Y47A)-N	ATGGAGAAATCGTCTGTGATGCCTTATTCTGTAAAGCATT
SaSsbC(Y47A)-C	CCAAATGCTTTACAGAATAAGGCATCACAGACGATTTCTC
SaSsbC(F53A)-N	GATTACTTATTCTGTAAAGCAGCTGGCAAGTTAGCTTCTA
SaSsbC(F53A)-C	TATTAGAAGCTAACTTGCCAGCTGCTTTACAGAATAAGTA
SaSsbC(Y81A)-N	GGTCAAATGAGATCAAGAAAGGCTGATAAAGACGGACAAA
SaSsbC(Y81A)-C	GTGTTTGTCCGTCTTTATCAGCCTTTCTTGATCTCATTTG

These plasmids were verified by DNA sequencing. Underlined nucleotides indicate the designated site for the restriction site or the mutation site.

### Protein expression and purification

Purification of the recombinant SaSsbA [30], SaDnaD [31], SaPriA [42], and KpSSB [36] has been reported. The recombinant SaSsbC was expressed and purified using the protocol described previously for PriB [51]. Briefly, *E. coli* BL21(DE3) cells were transformed with the expression vector and overexpression of the expression plasmids was induced by incubating with 1 mM isopropyl thiogalactopyranoside. The protein was purified from the soluble supernatant by Ni^2+^-affinity chromatography (HiTrap HP; GE Healthcare Bio-Sciences), eluted with Buffer A (20 mM Tris-HCl, 250 mM imidazole, and 0.5 M NaCl, pH 7.9), and dialyzed against Buffer B (20 mM HEPES and 100 mM NaCl, pH 7.0). Protein purity remained at >97% as determined by SDS-PAGE (Mini-PROTEAN Tetra System; Bio-Rad, CA, USA).

The recombinant tag-free SaSsbC was expressed and purified using the protocol described previously [53] for *Pseudomonas aeruginosa* SSB (PaSSB) and *Salmonella enterica* serovar Typhimurium LT2 SSB (StSSB) with the following modifications. The cells overexpressing the protein were chilled on ice, harvested by centrifugation, resuspended in Buffer C (20 mM Tris-HCl and 50 mM NaCl, pH 7.9) and disrupted by sonication with ice cooling. The protein solution (50 mL) was precipitated from the supernatant of the cell lysate by incubation with 0.27 g/mL of ammonium sulfate for 30 min and centrifugation at 20000 g for 10 min. The pellets were washed twice with 2.0 mL of Buffer D (20 mM Tris-HCl, 50 mM NaCl, and 1.2 M ammonium sulfate, pH 7.9). After dialysis against Buffer C, the protein solution applied to the Q column (GE Healthcare Bio-Sciences, Piscataway, NJ, USA) was eluted with a linear NaCl gradient from 0.1 to 0.6 M with Buffer C using the AKTA-FPLC system (GE Healthcare Bio-Sciences, Piscataway, NJ, USA). The peak fractions with the ssDNA binding activity were collected and dialyzed against Buffer E (20 mM potassium phosphate, 1 mM EDTA, and 100 mM NaCl, pH 7.0). The protein solution was then applied to the Heparin HP column (GE Healthcare Bio-Sciences, Piscataway, NJ, USA) and eluted with a linear NaCl gradient from 0.1 to 0.5 M with Buffer E. The peak fractions from this chromatographic step with the ssDNA binding activity were collected and concentrated. Protein purity of tag-free SaSsbC remained at >97% as determined by SDS-PAGE.

### Gel-filtration chromatography

Gel-filtration chromatography was carried out by the AKTA-FPLC system (GE Healthcare Bio-Sciences, Piscataway, NJ, USA). In brief, purified SaSsbC (2 mg/mL) in Buffer B was applied to a Superdex 200 prep grade column (GE Healthcare Bio-Sciences, Piscataway, NJ, USA) equilibrated with the same buffer. The proteins were detected by measuring the absorbance at 280 nm. The column was calibrated with proteins of known molecular weight: thyroglobulin (670 kDa), γ-globulin (158 kDa), albumin (67 kDa), ovalbumin (43 kDa), chymotrypsinogen A (25 kDa) and ribonuclease A (13.7 kDa).

### Electrophoretic mobility shift assay (EMSA)

EMSA for SaSsbC was conducted using the protocol described previously for SSB [33]. Briefly, radiolabeling of various lengths of ssDNA oligonucleotides was carried out with [γ^32^P]ATP (6000 Ci/mmol; PerkinElmer Life Sciences, Waltham, MA) and T4 polynucleotide kinase (Promega, Madison, WI, USA). The protein (0, 0.01, 0.02, 0.039, 0.078, 0.1563, 0.3125, 0.625, 1.25, and 2.5 μM; tetramer) was incubated for 30 min at 25° C with 1.7 nM DNA substrates in a total volume of 10 μL in 20 mM Tris-HCl pH 8.0 and 100 mM NaCl. Aliquots (5 μl) were removed from each of the reaction solutions, and added to 2 μl of gel-loading solution (0.25% bromophenol blue and 40% sucrose). The resulting samples were resolved on a native 8% polyacrylamide gel at 4° C in TBE buffer (89 mM Tris borate and 1 mM EDTA) for 1 h at 100 V, and were visualized by phosphorimaging. The phosphor storage plate was scanned, and the data for complex and free DNA bands were digitized for quantitative analysis. The ssDNA binding ability for the protein was estimated using linear interpolation from the protein concentration that binds 50% of the input DNA.

### Preparation of dsDNA substrate

The double-stranded DNA substrates (dsDNA) were prepared with a radiolabeled PS4 strand (3′-GGGCTTAAGCCTATCGAGCCATGGG-5′; 25 mer) and an unlabeled PS3 strand (5′-CCCGAATTCGGATAGCTCGGTACCC-3′) at a 1:1 concentration ratio. Unlabeled PS3-dT5, PS3-dT10, PS3-dT15, PS3-dT20, and PS3-dT25 strands were also used with PS4 for preparation of dsDNA substrates. Each dsDNA substrate was formed in 20 mM HEPES (pH 7.0) and 100 mM NaCl, by brief heating at 95° C for 5 min and then followed by slow cooling to room temperature overnight.

### Preparation of forked DNA substrate

The forked DNA substrate was prepared with a radiolabeled M2 strand (5′- AAGCTGTGGTGGTAACAAGTAGTGCCGGTGAAGCGGCGCACGAAAAACGCGAAAGCGTTTCACGATAAATGCGAAAAC-3′; 78 mer), an unlabeled M1 strand (5′- GTTTTCGCATTTATCGTGAAACGCTTTCGCGTTTTTCGTGCGCCGCTTCATGTACACCGTTCATCTGTCCTCGTTCAAAGTTGGTCAGTT-3′; 90 mer), and an unlabeled M3 strand (5′-CCGGCACTACTTGTTACCACCACAGCTT-3′; 28 mer) at a 1:1:1 concentration ratio. S1/M2-M3 forked DNA substrate was prepared with a radiolabeled M2 strand, an unlabeled S1 strand (5′-GTTTTCGCATTTATCGTGAAACGCTTTCGCGTTTTTCGTGCGCCGCTTCATGTACACCGTTCATCTGTCC-3′; 70 mer), and an unlabeled M3 strand at a 1:1:1 concentration ratio. These forked DNA substrates were formed in 20 mM HEPES (pH 7.0) and 100 mM NaCl, by brief heating at 95°C for 5 min and then followed by slow cooling to room temperature overnight.

### Chemical cross linking

The oligomerization state of SaSsbC was analyzed by chemical cross linking using glutaraldehyde. SaSsbC (2.5 μM) was incubated with increasing concentrations of glutaraldehyde (0.1% to 5%) at 4° C for 30 min. The reactions were stopped by adding SDS sample buffer and were fractionated on Coomassie Blue-stained SDS-PAGE.

### ATPase assay

SaPriA ATPase assay [31] was performed with 0.4 mM [γ-^32^P] ATP and 0.12 μM SaPriA in reaction buffer containing 40 mM Tris (pH 8.0), 10 mM NaCl, 2 mM DTT, 2.5 mM MgCl_2_, and 0.1 μM PS4/PS3-dT30 DNA substrate. Aliquots (5 μL) were taken and spotted onto a polyethyleneimine cellulose thin-layer chromatography plate, which was subsequently developed in 0.5 M formic acid and 0.25 M LiCl for 30 m. Reaction products were visualized by autoradiography and quantified with a phosphorimager.

### Site-directed mutagenesis

SaSsbC mutants were generated according to the QuikChange Site-Directed Mutagenesis kit protocol (Stratagene, LaJolla, CA, USA) using the primers (Table 3) and wild-type plasmid pET21b-SaSsbC as template. The presence of the mutation was verified by DNA sequencing.

### Bioinformatics

The amino acid sequences of 698 sequenced SSB homologs were aligned using ConSurf [32, 59]. The model of SaSsbC was built from the coordinates of 5XGT (crystal structure of SaSsbA) by using SWISS-MODEL, http://swissmodel.expasy.org/. The structures were visualized by using the program PyMol.
